# Hydration shell differentiates folded and disordered states of a Trp-cage miniprotein, allowing characterization of structural heterogeneity by wide-line NMR measurements

**DOI:** 10.1038/s41598-019-39121-5

**Published:** 2019-02-27

**Authors:** Nóra Taricska, Mónika Bokor, Dóra K. Menyhárd, Kálmán Tompa, András Perczel

**Affiliations:** 1 Laboratory of Structural Chemistry and Biology, Institute of Chemistry, Eötvös Loránd University, Budapest, 1117 Hungary; 2 MTA-ELTE Protein Modelling Research Group, Pázmány Péter st. 1A, 1117 Budapest, Hungary; 3 Institute for Solid State Physics and Optics, Wigner RCP of the HAS, 1121 Budapest, Hungary

## Abstract

Hydration properties of folded and unfolded/disordered miniproteins were monitored in frozen solutions by wide-line ^1^H-NMR. The amount of mobile water as function of *T* (−80 °C < *T* < 0 °C) was found characteristically different for folded (TC5b), semi-folded (pH < 3, TCb5(H+)) and disordered (TC5b_N1R) variants. Comparing results of wide-line ^1^H-NMR and molecular dynamics simulations we found that both the amount of mobile water surrounding proteins in ice, as well as their thaw profiles differs significantly as function of the compactness and conformational heterogeneity of their structure. We found that *(i*) at around −50 °C ~50 H_2_Os/protein melt *(ii*) if the protein is well-folded then this amount of mobile water remains quasi-constant up to −20 °C, *(iii*) if disordered then the quantity of the lubricating mobile water increases with *T* in a constant manner up to ~200 H_2_Os/protein by reaching −20 °C. Especially in the −55 °C ↔ −15 °C temperature range, wide-line ^1^H-NMR detects the heterogeneity of protein fold, providing the size of the hydration shell surrounding the accessible conformers at a given temperature. Results indicate that freezing of protein solutions proceeds by the gradual selection of the enthalpically most favored states that also minimize the number of bridging waters.

## Introduction

Polypeptide and protein drugs are becoming ever more common in the pharmaceutical industry (over 130 different products now approved for clinical use by FDA) but the purification, formulation and storage of such drugs is a continuous challenge. Both cooling to subzero temperatures and heating above physiological temperatures results in the unfolding of globular proteins, in a great variety of solvents^1,2^. While the loss of global fold at high temperatures has been studied in detail, the significance and mechanism of cold denaturing are still under debate^3–8^. One of the major difficulties of studying the process is ice formation, even though a few examples can be found where proteins manifest detectable unfolding already above water freezing temperatures^9,10^ – this, however is not the general case. Proteins frozen in ice undergo a dynamical transition when heated above ~−70 °C, leading to the functionally relevant forms with the simultaneous onset of water motion within the first hydration shell^11^, which has long been shown to be required for activity^12–14^. Without a free and mobile water shell, functionally important protein motions cannot take place and there is a strong coupling between protein and water dynamics which can only be uncoupled at cryogenic temperatures^15–17^. However, the extent of inter-connectedness of the two processes is still unclear.

Studies aimed at understanding the mechanism of cold denaturing are usually either theoretical, where accessible simulation times (and the shortcomings of the available water models) render ice-formation unlikely^18–20^ or experimental, where the application of pressure or additives interferes with ice formation^21–24^. Therefore, these studies disregard the interaction between the freezing/un-freezing of the hydration layer and of the solute protein molecules. It is now generally thought that during cooling – as opposed to heat denaturation – the protein 3D-fold preserves some of its compact native structure, resulting in a state better described as water-penetrated, than an unfolded one. The process seems to involve the extended hydration of both hydrophobic and hydrophilic residues, with most recent results pointing to the significance of the latter phenomena^10,18,25^.

In this work our aim was to deduce the structural consequences of freezing in case of similarly sized but conformationally very different proteins in the −80 °C to 0 °C temperature range, through monitoring their hydration properties by wide-line ^1^H-NMR spectroscopy without interfering with the freezing of their solution. The applied NMR technique relies on the detection and analysis of the limited but significant amount of water that remains mobile around proteins – hydration waters^14^ – way below 0 °C. This approach also allows for the experimental characterization of hindered-rotation barriers and mapping of the energetic heterogeneity of water molecules bound to the molecular surface of proteins^26,27^. We have previously studied larger proteins with this technique^26,27^ and have identified differing hydration properties of globular and intrinsically disordered proteins (IDPs). While in case of globular proteins, a wide temperature range exists where the amount of mobile water doesn’t change with increasing *T*, after melting of the first hydration layer of IDPs the thaw is continuous as *T* increases. The applied method allows for quantification of structural heterogeneity (the ratio of heterogeneous binding interface values (*HeR*)) based directly on the melting diagrams (MD) that report the amount of molten water measured by NMR as a function of temperature^26^.

TC5b, a miniprotein, was chosen as the test case for the present cold-temperature studies because by small changes in its sequence great structural alterations can be evoked. TC5b consists of 20 residues and folds autonomously (under 4 µs in water at neutral pH)^28^. It was produced - by truncation and designed mutations - from of Exendine-4^29^, a protein drug used as a key GLP-1 receptor agonist^30^ that potentiates insulin secretion in pancreatic β-cells^31,32^ and is used in the daily clinical practice to treat type II *Diabetes Mellitus*^33–36^. The TC5b shortened variant has a well-defined 3D structure under physiological conditions: a hydrophobic core clustered around Trp^6^, surrounded by multiple secondary structural elements (α-helix, β-turn and polyprolin-II helix). TC5b has a cooperative melting profile with a protein like melting temperature: (~40–50 °C) with folding propensities like those of globular proteins. Its internal dynamics and folding pathways were studied in detail^28,29,37–44^. The Asn^1^→Arg switch in its sequence (TC5b_N1R) leads from folded to a largely unfolded state (Table 1)^45^. Shifting the pH below 3 (TC5b(H+)) results in the appearance of partly-folded I-states^37,38,46^ characterized by elevated internal dynamics. Thus, three systems of nearly identical chemical composure representing three very different classes of proteins’ fold (Folded (F)-, Intermediate (I)- and Unfolded (U)-states) could be studied and compared directly. Besides being easy to tune, these proteins are small enough to be characterized thoroughly by molecular dynamics simulations (MDSs) which allow, for the first time, enhancement of the NMR results of ice trapped macromolecules with atomistic detail.

**Table 1:** Amino acid sequences and net-charges of (mini)proteins studied by wide-line NMR.

pH = 7		pH < 3	
Sequence and localized charges	net charge	Sequence and localized charges	net charge
**TC5b: = **H^+^_2_−**N**LYIQWLK^+^D^−^GGPSSGR^+^PPPS-O^−^	+1 (+0.9)^*a*^	**TC5b**(H+):** = **H^+^_2_−**N**LYIQWLK^+^D^(0)^GGPSSGR^+^PPPS-O^(½−)^	+2.5 (>2.5)^*a*^
**TC5b_N1R: = **H^+^_2_-R^+^LYIQWLK^+^D^−^GGPSSGR^+^PPPS-O^−^	+2 (+1.9)^*a*^	**TC5b_N1R**(H+):** = **H^+^_2_−R^+^LYIQWLK^+^D^(0)^GGPSSGR^+^PPPS-O^(½−)^	+3.5 (>3.5)^*a*^

^*a*^Calculated by PROTEIN CALCULATOR v3.4.

## Results and Discussion

**Figure 1:**
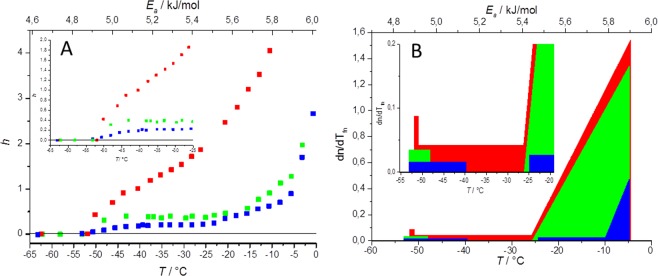
(**A**) Melting diagram – where *h* is the mass-normalized extent of hydration (in g water/ g protein units) - in TC5b in neutral (green) and acidic condition (blue) as well as in case of TC5b_N1R dissolved in water at pH = 6.9 (red). (**B**) Derivative melting diagram of TC5b in neutral (green) and acidic condition (blue) as well as that of the TC5b_N1R dissolved in water at pH = 6.9 (red).

**Figure 2:**
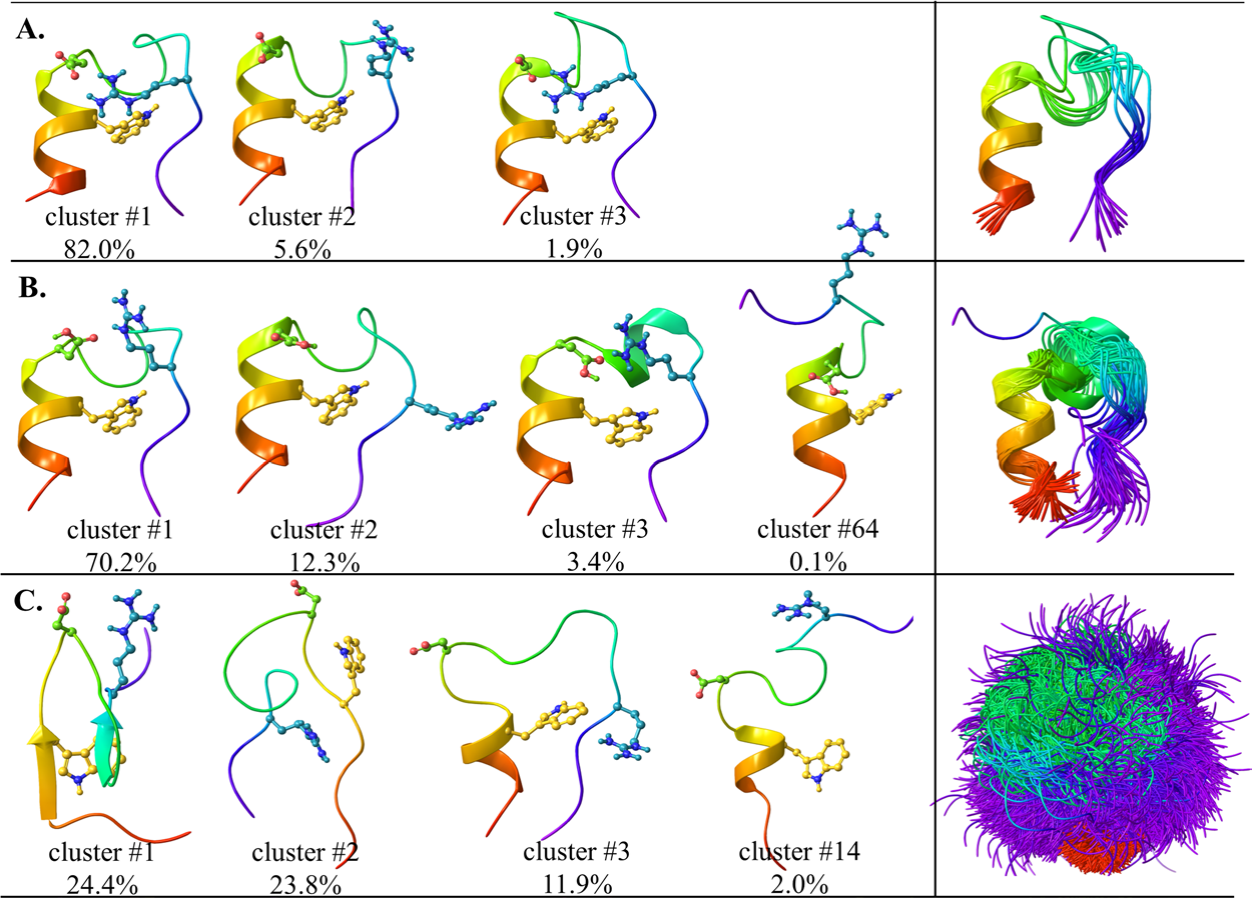
Left side: Characteristic backbone scaffolds of TC5b (**A**), TC5b(H+) (**B**) and TC5b_N1R (**C**) (showing Trp^6^ (in yellow), Asp^9^ (in green) and Arg^16^ (in blue) explicitly. Right side: Superimposed mid-structures of the most populated clusters (using a 1 Å cutoff) accounting for at least 90% of all snapshots of the last 800 ns of the molecular dynamics simulations.

TC5b has a compact, well-folded structure. Preservation of the folded, globular state of TC5b (neutral conditions) – even at low temperatures – was reflected by our wide-line NMR measurements (Fig. 1). Heating of the frozen sample from −80 °C affords a melting diagram (MD) characteristic for well folded proteins: after the thaw of first hydration layer (*T* = −53 ± 1 °C, *E*_*a*_ = 4.85 ± 0.02 kJ/mol), a plateau can be seen in the −48 ± 1 °C ≤ *T* ≤ −26 ± 1 °C temperature range (corresponding (as described in the experimental section) to a 5.01 ± 0.02–5.44 ± 0.02 kJ/mol potential energy range Figs 1 and 2S) indicating a temperature region where no additional water molecules melt. This means that the potential energy barriers corresponding to this temperature range are insurmountable for further waters present in the system. In other words, the protein and its lubricating shell of unfrozen waters remains intact; the energy of heating is absorbed in form of enhanced motions of these hydration water molecules. The melting of the first hydration layer at ~−50 °C (4.92 ± 0.02 kJ/mol) is in good agreement with our previous experience concerning a great variety of proteins^15,16,26,27^, but also with the temperature where the water molecules specifically bound to the 2D surface of a polymer (with regularly spaced –OH groups to model solvated protein surfaces) start percolation – spreading – on the surface from previously formed low density water patches^47^. This is also the temperature range where the movement of water associated to the protein surface starts in wetted powder samples^11^ and where molecular oxygen trapped in the hydration shell of hemoglobin is liberated to diffuse into the protein matrix^17^.

The number of waters that become mobile during the first thaw can be estimated using the *n* value derived directly from the FID signal. Here, this corresponds to approximately 50 water molecules. Comparing this value to the MDS derived solvent structure (see Fig. 4S) we can conclude that only waters very close to the protein surface (within 2.6 Å) melt during this first melting step.

From *T* = −26 ± 1 °C, the amount of melted water continuously rises as temperature increases. This steep final section of the melting diagram (5.45 ± 0.02–5.90 ± 0.02 kJ/mol) can be attributed to water molecules in energetically heterogeneous interactions with the accessible protein residues of the surface (Fig. 2S).

**Figure 3:**
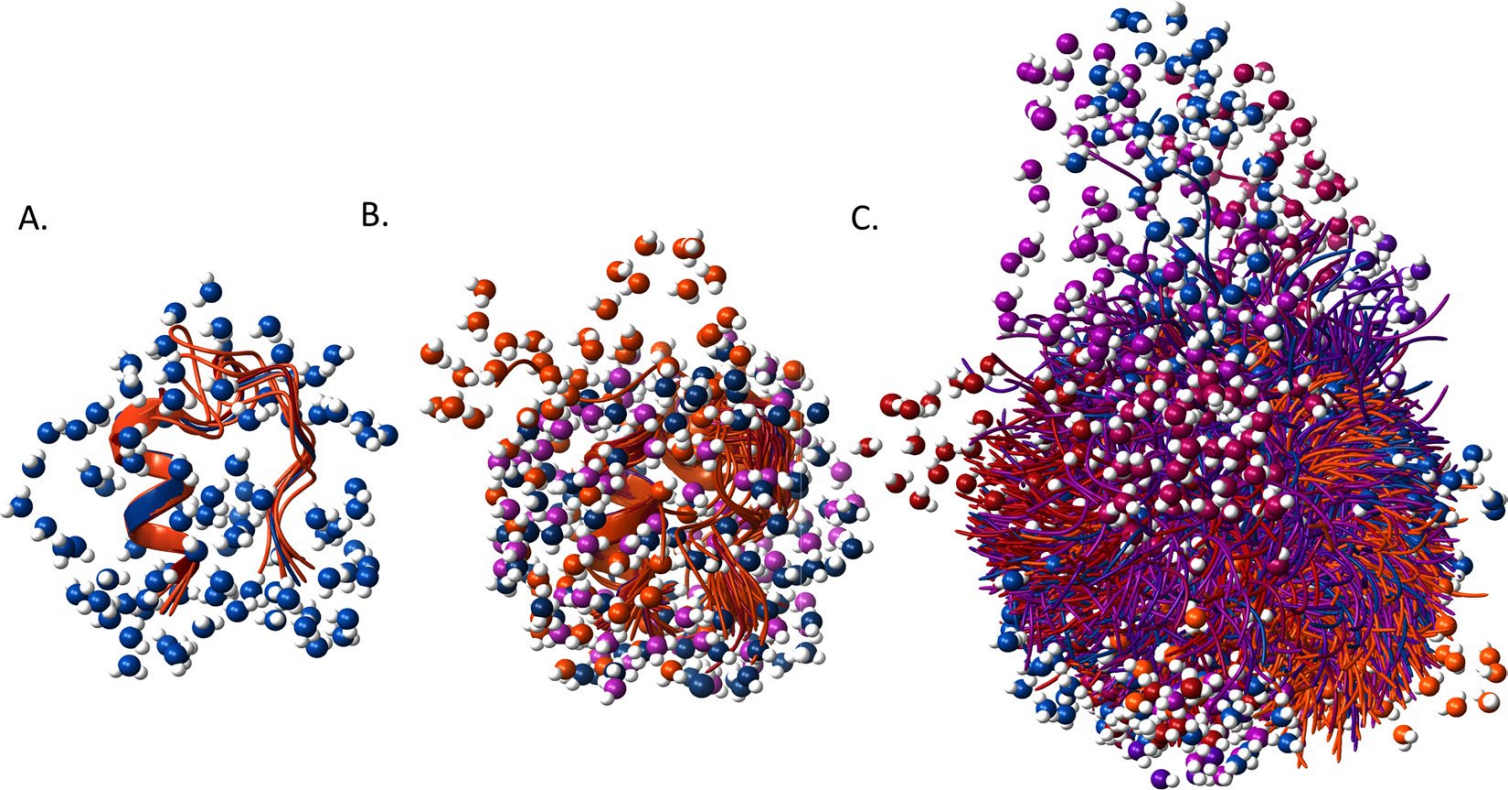
Backbone structures of TC5b (**A**), TC5b(H+) (**B**) and TC5b_N1R (**C**) conformers accounting for at least 90% of all snapshots of the last 800 ns of the molecular dynamics simulations with surrounding surface waters within 2.8 Å of the protein surface. Conformers are colored from blue to orange - with blue showing the most populated clusters’ mid-structures, while lighter colors indicating those of the less populated clusters’. Liquid H_2_Os are colored in line with the color of the backbone scaffold. For a more detailed analysis of the water-structure, see Fig. 4S.

MDS also indicated that the fold of neutral TC5b is composed of nearly homogenous and conserved scaffolds over time, as expected (Fig. 3S)^29,38^. Grouped based on their backbone structural similarities, the mid-structure of the most populated cluster (cluster #1), accounting for over 82% of the snapshots, could be fit with a 0.7 Å rmsd to the backbone of the NMR determined solution state structure (PDB 1l2y) (Fig. 2), also confirming the applicability of the presently used MDS-protocol.

It was shown in previous ambient temperature solution-state NMR measurements that under acidic conditions (affording TC5b(H+)), local charge shifts (Table 1) of TC5b(H+) result in the loss of the key fold stabilizing salt-bridge of Asp^9^-Arg^16^ as the Asp side chain becomes protonated^46^ leading to a dynamic ensemble of several semi-folded I-states. In the present wide-line NMR measurements we found that below pH = 3 the thaw of first hydration layer of TC5b(H+) becomes more extended, taking place in the temperature range of −53 ± 1 °C ≤ *T* ≤ −40 ± 1 °C (corresponding to 4.82 ± 0.02–5.13 ± 0.02 kJ/mol potential energy barriers). This is followed by a temperature independent plateau (−40 ± 1 °C ≤ *T* ≤ −25 ± 1 °C) (Fig. 1) where the thaw stops and no further waters can be mobilized by increasing temperature (or the available potential energy). At pH < 3, the side chain of Asp is dominantly (>90%) neutralized Asp^(0)^ (with a *C*′-terminal of main chain being half-protonated/deprotonated (50%**/**50%) (Table 1)), so MDS of TC5b was repeated with a protonated Asp^9^ to describe TC5b(H+). The folded-like structure and the α-helical segment was conserved, however in only 12% of the structures we did find an H-bond between Asp^9^ and Arg^16^, as opposed to the >92% of the structures of the TC5b simulation (Fig. 2). In spite of this, the most populated cluster of TC5b(H+) (cluster #1), encompassing 70.2% of all structures, comprises globularly folded forms, similar to that of TC5b (backbone rmsd: 0.6 Å). Furthermore, cluster #2 of TC5b(H+) (12.3% of the structures) also shows quite a similar 3D-fold, with slightly more opened backbone structures. However, elements of the above 2 clusters are less stringently packed compared to those of TC5b(neutral): the relative (average) backbone mobility increased by a factor of 1.9 and 2.2, in case of clusters #1 and #2 of TC5b(H+), as compared to that of cluster #1 of TC5b. The sampling of such loosened arrangements potentiates various unfolding routes resulting in the appearance of conformers where – for example – the 3_10_-helix of residues 10–14 is turned into an α-helix (3% of the structures), or where the Trp-cage opens up completely (0.1%) (Fig. 2). These findings allow elaboration of the melting profile: because of the different conformers contributing to the “folded” ensemble which all possess a unique hydration layer, the first melting event is prolonged, and due to the higher number of low energy open forms, the temperature independent plateau (−40 ± 1 °C ≤ *T* ≤ −25 ± 1 °C or 5.13 ± 0.02 kJ/mol ≤ *E*_a_ ≤ 5.46 ± 0.02 kJ/mol) is shortened (Figs 1 and 2S) but is still present and characteristic. This means that at pH < 3, the TC5b carries some characteristics of both folded and disordered proteins.

Although TC5b_N1R differs from TC5b only by its first amino acid, this mutation has a large and global conformational effect. In principle, the side chain of the positively charged *N*-terminal Arg(+) could hook its *C*-terminal *via* an Arg^1(+)^ ↔ -COO^(−)^ salt bridge, but this does not take place because the α-helix macro-dipole clashes with the positive charge of Arg^1^ and the backbone unfolds into a highly dynamic U-state^45^. In line with this, the MDS derived equilibrium structural ensemble of TC5b_N1R consists of greatly varied/perturbed conformers. Only about ~12% of the snapshots have a folded structure (Fig. 2), the remaining 88% forms the ensemble of a U-state. Accordingly, melting of the hydrate layer as recorded by wide-line NMR is continuous (Figs 1 and 2S) once the melting process starts (at −50 ± 1 °C or 4.91 ± 0.02 kJ/mol), and from this point the number of hydration water molecules that become mobile around the protein increases at a constant rate with increasing temperature, which results in a constant derivate in the 4.88 ± 0.02–5.45 ± 0.02 kJ/mol energy region. The *n* value of the initial thaw signals the presence of compact conformers lubricated by ~50 water molecules, similarly to that seen in case of TC5b. As heating progresses, more and more H_2_Os become mobile, clear characteristics of an IDP. At −35 °C (5.24 ± 0.02 kJ/mol; about the middle of the plateaus of TC5b and TC5b(H+)) there are more than 120 mobile water/protein present, thus more than twice as many as in its more ordered counterparts.

We found that to explain the basic features of the observed melting curves it is sufficient to consider the room temperature backbone-conformational heterogeneity of the studied proteins. Using a quite strict cutoff value of 1 Å, 90% of all snapshots of the equilibrated MDS trajectory could be adequately described using the 10 most populated conformers of TC5b (cluster #1 of the folded form accounts for >80% of them). On the contrary, 73 clusters for the loose, but still folded TC5b(H+) (cluster #1 accounting for >70% of them) and as many as 6042 clusters are required for depicting the conformational ensemble of TC5b_N1R. For the latter molecule, the most folded-like cluster comprises only 12% of all structures (Fig. 2). The three systems studied here therefore represent three thermodynamically different states: (*i*) TC5b has a practically non-degenerate F-state and very high energy unfolded region. (*ii*) The folded state of TC5b(H+) is moderately degenerate while its unfolded region remains high in energy and scarcely populated. (*iii*) For TC5b_N1R the folded and unfolded ensembles overlap in energy and are degenerate.

These findings are in correlation with the ratio of heterogeneous binding interface values (*HeR*) derived from the wide-line NMR measured MDs (Table 2): 0.536 ± 0.007, 0.687 ± 0.003 and 1 for TC5b, TC5b(H+) and TC5b_N1R, respectively, also indicating a TC5b > TC5b(H+) >> TC5b_N1R order of backbone scaffold homogeneity. The surface of TC5b is partly homogeneous (indicated also by zero derivative section) and partly (~54%) heterogeneous, probably due to the highly flexible C-terminal region of the protein. In case of TC5b(H+) the heterogeneous content increases (~69%) and TC5b_N1R is fully disordered (100%) based on this descriptor too.

**Table 2:** The determined *T*_fno_, *T*_fne_ and *HeR* of three miniproteins.

	*T*_fno_ − *T*/°C	*T*_fne_ − *T*/°C	*HeR*
TC5b	0.842 ± 0.008 −43 ± 1 °C	0.915 ± 0.008 −23 ± 1 °C	0.536 ± 0.007
TC5b(H+)	0.850 ± 0.008 −41 ± 1 °C	0.897 ± 0.008 −28 ± 1 °C	0.687 ± 0.003
TC5b_N1R	—	—	1

*T*_fno_ and *T*_fne_ are the beginning and end point of the d*n*/d*T*_fn_ ~ 0 plateau region.

The ratio of heterogeneous binding interface (*HeR*) was defined as ${\boldsymbol{HeR}}{\boldsymbol{=}}({\bf{1}}\,{\boldsymbol{-}}\,{{\boldsymbol{T}}}_{\text{fne}})/({\bf{1}}\,{\boldsymbol{-}}\,{{\boldsymbol{T}}}_{\text{fno}})$.

**Figure 4:**
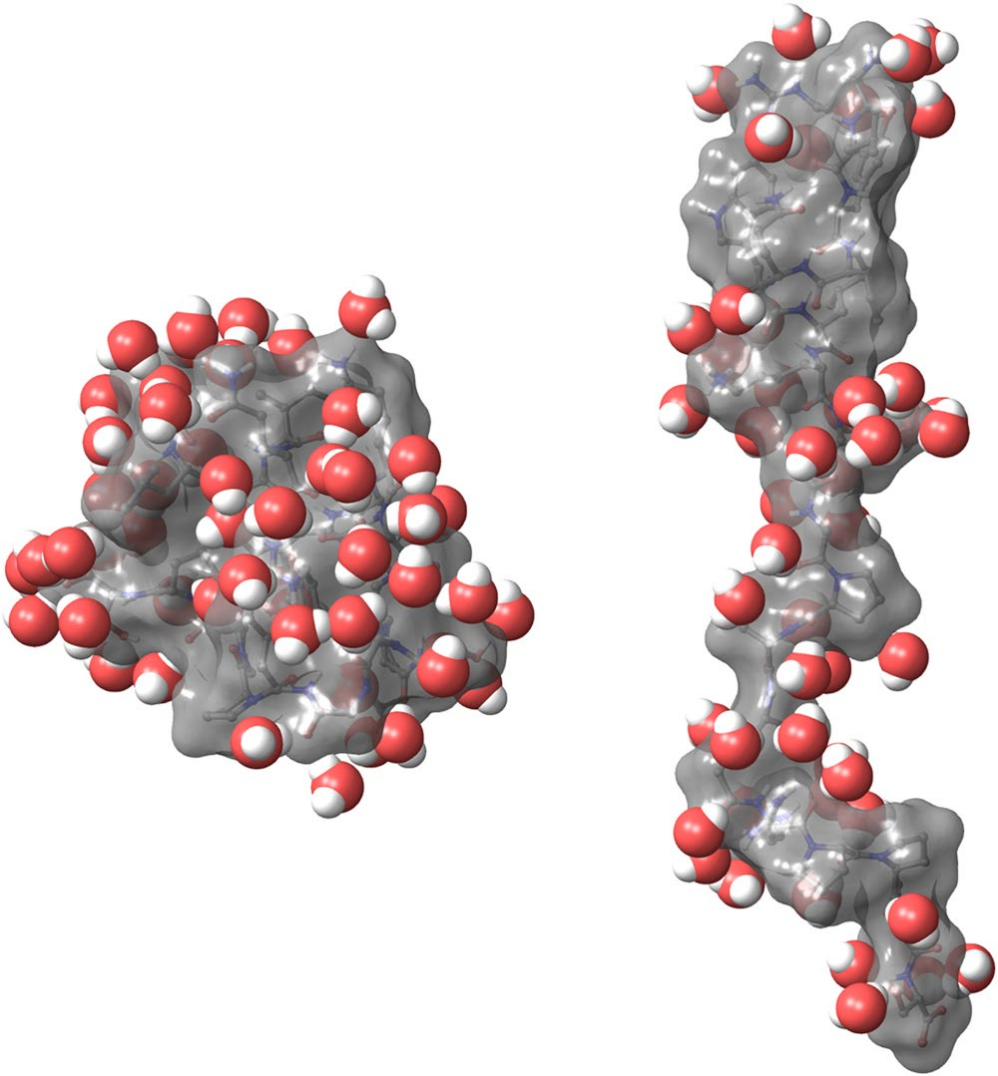
The closest 50 water molecules solvating a folded conformation of TC5b (left) and an unfolded conformation of TC5b_N1R (right) (snapshots from MDS).

With all the above considered, we can provide a “high resolution” picture of the melting process of these proteins starting from their encapsulated forms in ice at −80 °C (*E*_*a*_ = 4.26 ± 0.02 kJ/mol). Approximately 30–70 water molecules per protein melt at ~−50 °C (*E*_*a*_ = 4.92 ± 0.02 kJ/mol) (determined from the three MDs) and start protein surface lubrication first (see Fig. 4S). This amount of H_2_O corresponds to the first hydration layer, within 2.5–2.8 Å of the protein surface. As these waters must bridge the irregular surface of the molecular scaffolds and the edges of ordered bulk ice, the amount of melted water is expected to be chiefly determined by the size of the solvent accessible surface of the conformers that are populated at a given temperature. Sequences that do not possess a dominant fold but comprise instead of a multitude of conformationally versatile and easily accessible backbone scaffolds, when heated from low temperatures will gradually populate all these states. Appearance of structurally distinct protein folds loosens the surrounding structure of the ice, since the gradual increase of the available thermal activation energy as *T* increases will allow for the melting of the hydration shell of less and less compact conformers, with growing number of mobile waters, as is the case of TC5b_N1R (Fig. 3).

In contrast to this, for a well-structured globular protein with no accessible low-lying states, after the melting of the first hydration shell, heating will only enhance the mobility of both the trapped protein and the liberated waters but for a considerable temperature range (5.01 ± 0.02–5.44 ± 0.02 kJ/mol) no increase in the number of freely-moving waters will be seen: here the rate of the introduction of newly mobilized water molecules and thus the derivative of the melting diagram (d*n*/d*T*_fn_) is zero (Figs 1 and 2S). In other words, the accessible potential energies in this temperature range are insufficient for inducing the transfer of any of the waters present from ice-contained to the melted state. This “zero-derivative” region is the most prominent feature of the melting diagram of TC5b at neutral pH. TC5b(H+) presents an example of the intermediate between the two extremes, having few low-lying states which provide a continual increase of freely-moving waters during the first melting thaws starting at ~−50 °C (4.92 ± 0.02 kJ/mol), but after this, *n*(*T*_fn_) forms a plateau (5.13 ± 0.02–5.43 ± 0.02 kJ/mol) and d*n*/d*T*_fn_ = 0. Clearly, these melting diagrams reflect on the conformational and energetic heterogeneity of the solvent accessible surface of the studied proteins.

Analyzing the first thaw of the melting process, we see that the amount of water liberated in this step is only enough for “covering” compact – well-folded – conformers of the protein but it is simply too few to create a closed solvent layer of even monomolecular thickness around an open – unfolded – conformation (Fig. 4). Thus, it seems that special hydration effects attributed to cold denaturing lose some of their significance in the presence of ice formation, where, according to our results, cooling results in the gradual selection of the enthalpically most favored state with as few bridging waters as possible (as also illustrated by the continual decrease of *n* with decreasing temperatures).

Cold denaturing of TC5b was the subject of a detailed replica exchange molecular dynamics study published recently^18^, which offers an interesting comparison with the present results. Authors found distinct signs of cold denaturing below −60 °C (*E*_*a*_ = 4.70 ± 0.02 kJ/mol), remarkably close to the temperature range where our studies and those of others^11,47^ indicate the universal (heterogeneity independent) unfreezing of the first hydration shell. Cold denaturing was accompanied by *(i*) the widening of the Trp-cage (increase of the Trp^6^ ↔ Ser^14^ distance), *(ii*) loss of the Asp^9^ ↔ Arg^16^ salt bridge, *(iii*) increase in the number of protein-water H-bonds and *iv*) decrease of intramolecular contacts. However, all these changes have resulted in neither the significant increase of the solvent accessible surface area (SASA) nor that of the radius of gyration (*R*_g_). This supports the concept that cold denaturing results in loosening of intramolecular interactions but not the vast unfolding of the protein scaffold. Heat denaturing, on the other hand, lead to characteristic increase of both SASA and *R*_*g*_, with the reduction of 3D-fold dependent pairwise interactions. Similar tendencies could be captured at the room temperature MDSs of our three model systems, with TC5b(H+) behaving very similarly to the cold denatured state of TC5b, while TC5b_N1R corresponding to the heat denatured ensemble of TC5b (Fig. 5S). In case of TC5b(H+), beside the conformers where Ser^14^ points toward Trp^6^ and thus the inner region of the cage, other arrangements with similar backbone trace appear. Ser^14^ being immersed in the solvent allows for water to penetrate the hydrophobic core of the folded protein. The typical number of H-bonds between Asp^9^ and Arg^16^ is reduced from 2 (seen in case of TC5b) to 0. Since the *R*_*g*_ does not increase appreciably (the fold remains intact), the slight increase in SASA can be attributed to the moderate increase in the hydration of the inner domains of the folded form. In contrast, in the simulation of TC5b_N1R, the small Trp^6^ ↔ Ser^14^ distances belonging to the folded scaffolds were sparsely sampled. Instead an almost uniform distribution of longer distances resulted, reflecting the presence of a multitude of differently unfolded conformations. Interestingly, while the typical number of H-bonds between Asp^9^ and Arg^16^ is 0 in this case too, in ~20% of all the structures, well-oriented interaction and the formation of 2 H-bonds is seen. This, however, isn’t sufficient for the re-instatement of a folded scaffold. Broad distributions and a characteristic increase in the average values of both SASA and the *R*_*g*_ were seen, indicative of the presence of only a few really compact conformers in the equilibrium ensemble.

In the cold denaturing study of TC5b^18^ local solvent density fluctuation (commonly used to characterize the effective hydrophobicity of each amino acid residue) was also analyzed, and it was shown that at colder temperatures hydrophobic residues of the Trp-cage experience a pronounced decrease in hydrophobicity, a phenomenon measured by the decrease of the number of fluctuating H_2_Os of close proximity of a selected residue. Although the hydrophobic nature of Trp^6^, Gly^11^ and Pro^18^ is confirmed by the low number of waters associated with these residues during the present MDSs of TC5b, TC5b(H^+^) and TC5b_N1R too, fluctuation of H_2_Os near the hydrophobic core did not decrease in case of TC5b(H^+^). A slight increase was seen, which however is in agreement with the fact that backbone fold conformational heterogeneity also increases in this region – an effect that is not expected in cold temperature simulations (Fig. 6S).

The similarities of the two sets of MDSs indicate that hydrophilic residues and changes in their interactions must contribute greatly to the processes of cold denaturation and heat induced unfolding. It was clearly demonstrated that turning off the Asp^9^ ↔ Arg^16^ interaction by a pH shift initiates changes that resemble those of the cold denaturation of TC5b, while disturbing the electrostatics of the *N’*-termini leads to an unfolding similar to that experienced upon heating.

## Conclusion/Outlook

Our results indicate that basic features of a protein fold are not necessarily altered by freezing its solution. We showed that monitoring the melting process of frozen protein solutions by wide-line NMR spectroscopy affords an adequate description and a quantitative measure (*HeR*) for the heterogeneity of F-, I- and U-state proteins, and presents a clear-cut method for differentiating IDPs from well folded proteins and also from those systems that simply possess a native folded state that is degenerate. The comparison of the present experimental and MDS data with the results of cold denaturing studies^18^ shows that the loosening of tightly packed structures of the native fold and water penetration into the hydrophobic core is prompted most likely by the loss of hydrophilic intramolecular interactions. By cooling and heating the system or by the chemical inactivation of certain crucial charge-mediated interactions, very similar structural changes can be induced. Results also indicate that the transition of proteins from “frozen” to functional form should be expected above ~−55 °C, where mobile waters of the first hydration shell allow for the start of functionally relevant protein movements too.

## Methods

### Protein expression and purification

The measured miniproteins were produced by bacterial expression using previous published protocol^48^.

### Wide-line NMR spectroscopy

^1^H-NMR measurements and data acquisition were accomplished by a Bruker AVANCE III spectrometer at *ω*_0_/2π = 82.4 MHz with a stability of better than ±10^−6^, and with 2 ppm inhomogeneity of the magnet. The value represents the stability of the electromagnet, without any drift during measurements; the stability of the frequency is by two orders of magnitude better for our system. The recycle delay was 5*T_1_ and *T*_1_ was measured at various temperatures. The temperature was controlled by an open-cycle Janis cryostat, stable to ±0.1 °C and the uncertainty of the scale was ±1 °C. Data points are based on spectra recorded by averaging signals to reach a signal/noise ratio better than 50. The portion of mobile proton (water) fraction, *n*, is directly determined by the FID signal based on the comparison of the signal intensity extrapolated to zero time with the corresponding values measured at a temperature where the whole sample is in liquid state^49,50^. This procedure was shown to give identical results to those derived from CPMG-echo experiments for a variety of different proteins^16^. The number of averaged NMR signals was varied to achieve the desired signal quantity for each sample and for unfrozen water quantities. The typical length of the π/2 pulse was 2.2 µs. The sample volume was 70 µl. The sample tube is a cylindrical, capped Teflon capsule of 5 mm diameter. We use a probe designed for wide-line NMR with a simple coil.

### Data extraction, scaling and analysis

The chemical shifts or their evolution cannot be seen, they are buried by the width of the signal detected by wide-line NMR. All of the spins are on resonance as wide-line NMR can see them. The amplitude of the NMR response is directly proportional to the equilibrium state nuclear magnetization *M*_*i0*_ and so, to the number of contributing nuclei, *n*_i_ in the *i*^th^ nuclear spin fraction in the sample. The free induction decay (FID) signal can be separated into distinct fractions for a multicomponent system. The slowly decaying component is generated by the mobile ^1^H nuclei (mostly in hydration water molecules in our case). The extrapolated amplitude of a component corresponds to the magnetization induced by the involved nuclei as *M*_*i0*_ = *n*_i_*B*_0_/*T*. This expression gives the direct measure of each ^1^H fraction. In aqueous solution samples there are various water phases^51^ which freeze at lower temperature *i.e*. well below 0 °C. The on-resonance measured FID (^1^H-NMR signal) at each temperature (−80 °C < *T* < 0 °C) comprises proton resonances decaying at different spin-spin relaxation rates, *T*_2_s, determined by their physico-chemical nature^52^. Protons of the ice and the protein relax faster (*T*_2_ < 50–100 μs) compared to those of freely moving liquid water (*T*_2_ > 100 µs) (Fig. 1S). Thus, the amount of ^1^Hs forming the FID could be clustered and separated based on their relaxation properties. So the signal detected in the time interval from zero to ∼100 μs is the mixture of transient signals over the dead-time period (6–8 µs) of the spectrometer and parts of ice and protein FID signals and in the analysis we used the signal of the moving water which is a protractedly decaying signal (between 200 μs and 1 ms). In the previous study^27^ we could demonstrate the absence of this signal, where we measured the protein sample after vacuum drying. In this case only the quickly decaying protein signal was detected without water protons.

In this article the measured FID signals are converted into the portion of mobile proton (water) fraction, *n*. The decays of the mobile elements are not exponential due to the inhomogeneity of the external magnetic field. We fitted Gaussian functions to the FID data points at each temperature and used the coefficient of slowly relaxing protons (200 μs – 1 ms). After normalization of *n* at every temperature with all mobile water (the value is 100% above 0 °C) first we determined the normalized fundamental temperature (*T*_fn_ = *T*/273.15 K) and then the potential barrier (*E*_a_(0 °C) = 6.01 kJ/mol) which energy is necessary for excitation of movement of water molecules. (The normalization of the energy scale is made by the melting heat of ice^53^.)

*T*_fno_ and *T*_fne_ are the beginning and end point of the d*n*/d*T*_fn_ ~0 plateau region. *T*_fno_ << *T*_fne_ is indicative of a folded protein (a protein in its F-state), while *T*_fno_ ≈ *T*_fne_ refers to a disordered one (a protein in its U-state). Based on this, the ratio of heterogeneous binding interface (*HeR*) was defined as ${HeR}=(1-{T}_{\text{fne}})/(1-{T}_{\text{fno}})$. For details see *Supporting information*. Using *n* and the concentration of the samples (corresponding roughly to the number of protein molecules/water molecules), the number of mobile waters at each temperature can be estimated. We converted the measured signal intensities *n* = *N*_*i*_/*N*_0_ into hydration as *h* = *n*·*m*_water_/*m*_protein_, where *N*_0_ is the signal intensity measured above 0 °C, *m*_water_ is the mass of water, and *m*_protein_ is the mass of protein.

### Molecular dynamics simulations

MDSs concerning both TC5b under neutral and acidic conditions of and TC5b_N1R were carried out as implemented in GROMACS^54^, using the AMBER99SB-ILDNP forcefield^55^. Starting conformers of all three models were derived from that of TC5b as determined by NMR (PDB 1l2y) with identical backbone structure. Systems were solvated by TIP3P water molecules in dodecahedral boxes with 10 Å buffer. The total charge of the system was neutralized and physiological salt concentration set using Na^+^ and Cl^−^ ions. Energy minimization of starting structures was followed by sequential relaxation of constraints on protein atoms in three steps (all of 100 ps). Trajectories of 2000–3000 ns NPT simulations at 325 K and 1 bar were recorded for further analysis (collecting snapshots at every 4 ps). Clustering of conformations^56^ was carried out based on the main-chain conformation of snapshots of the last 800 ns of the simulations using a cutoff of 1.0 Å. Waters near the surface of the protein were counted using the select command of GROMACS^54^.

## Supplementary information

Hydration shell differentiates folded and disordered states of a Trp-cage miniprotein, allowing characterization of structural heterogeneity by wide-line NMR measurements
