# Supplementary material for: Hydration shell differentiates folded and disordered states of a Trp-cage miniprotein, allowing characterization of structural heterogeneity by wide-line NMR measurements
Source: Sci Rep. 2019 Feb 27;9:2947. doi: 10.1038/s41598-019-39121-5 (PMC6393587; doi:10.1038/s41598-019-39121-5)
Supplement: Supplementary file 1 — Hydration shell differentiates folded and disordered states of a Trp-cage miniprotein, allowing characterization of structural heterogeneity by wide-line NMR measurements [file 41598_2019_39121_MOESM1_ESM.pdf]

Supporting Information

Hydration shell differentiates folded and disordered states of a Trp-cage miniprotein, allowing characterization of structural heterogeneity by wide-line NMR measurements

Nóra Taricska[1], Mónika Bokor[3], Dóra K. Menyhárd[2], Kálmán Tompa[3] and András Perczel[1,2,*]

[1]Laboratory of Structural Chemistry and Biology, Institute of Chemistry, Eötvös Loránd University
[2]MTA-ELTE Protein Modelling Research Group, Pázmány Péter st. 1A, H-1117 Budapest, Hungary
[3]Institute for Solid State Physics and Optics, Wigner RCP of the HAS, H- 1121 Budapest, Hungary
* perczel@chem.elte.hu

Preparation of the NMR-samples: The lyophilized miniproteins were dissolved in distilled water. When proteins were measured in acidic conditions lyophilized proteins were used directly. (pH < 3 of HPLC purified proteins due to residual TFA is acidic.) To obtain pH neutral samples 1 mM NaOH was used (Radelkis OP-115 digital pH meter). Protein solution concentrations were determined by Jasco V-660 UV-Vis Spectrophotometer at 280 nm and found to be in the range of 1.2-1.9 mM.

^{1}H wide-line NMR: Hydrogen nuclei in water molecules, which are bound also to proteins, map the potential surface of the protein. The start of the motion of water molecules at low temperatures is detected by wide line ^{1}H-NMR. The mobile water molecules are found in the protein-solvent interfacial region, *i.e.*, at the surface of the protein molecules. With this technique it is possible to distinguish proton signals from different components (ice, water and protein) **Figure 1S**. In the previous study [1] we measured vacuum dried protein sample and in this case the water signal disappeared – only the narrow protein signal was detected. The width of the narrow spectrum component corresponding to the mobile ^{1}H nuclei is well below 1 kHz [2]. The reorienting side chain protons would give a much wider spectrum and therefore much shorter decay. Melting in the frozen protein solutions is considered as the appearance of a motionally narrowed component in the NMR spectrum, attributed to mobile water molecules [1-6]. The melting diagram (*MD*) gives the amount of molten water measured by NMR as a function of temperature. As an energy based temperature scale, the fundamental temperature, T_f is used. It can be formulated as $T_f = R{\cdot}T = k_B{\cdot}T{\cdot}N_A$ by definition* [7,8]. Furthermore, this is normalized to the melting point of ice as $T_{fn} = T/273.15$ K. The resulting normalized fundamental temperature, T_{fn} is then used for the sake of dimensionless units. T_{fn} is converted into energy units, by using the heat of melting of ice [9] as scaling factor $E_a = 6.01\ \mathrm{kJ{\cdot}mol^{-1}}{\cdot}T_{fn}$. The amount mobile, *i.e.* molten water is measured directly as a fraction of the total water content of the protein solution, *n* vs. *T*. *MD* can be formally described as a series expansion, $n(T_{fn}) = A+B(T_{fn}- T_{fne})+C(T_{fn}- T_{fn2})^2+D(T_{fn}- T_{fn3})^3+\ldots$, where the summation is carried out up to the cubic term [5,6]. The lowest temperature where mobile water molecules are detected is denoted by T_{fno}. The parameter T_{fne} gives the temperature where the thermal trend of the MD switches between constant and linearly increasing. Likewise, the trend changes from linear to quadratic at T_{fn2} and it becomes cubic at T_{fn3}. The quantities T_{fno} and T_{fne} can be read directly from the MD. The fitting procedure is carried out in sections. It starts with the lowest temperatures. If there is a constant *n* region, than the values of *A* is determined and kept for further analysis. Then follows the linear section and parameters *B* and T_{fne} are gained and used in sections of higher powers. The next section is increasing quadratically and *C* and T_{fn2} is fitted, then the cubic section is next with *D* and T_{fn3}. The distribution of the potential barriers involved in the mobility of hydration water molecules can be studied by using *DMD*s (derivative melting diagram). The fitted analytical expression for *n* is used to get DMD with differentiation. The change in *MD* with T_{fn} is given by the DMD as $\mathrm{d}n/\mathrm{d}T_{fn} = B+2C(T_{fn}- T_{fn2})+3D(T_{fn}- T_{fn3})^2+\ldots$ This is the number of the water molecules just mobilized at the actual temperature. Dynamic characteristics have been introduced to describe quantitatively the disordered or ordered state of protein molecules. These dynamic characteristics have significance beyond the static structural description. The heterogeneity ratio, $HeR = (1-T_{fne})/(1-T_{fno})$ gives the ratio of the heterogeneous binding interface and it is a measure of the structural disorder of proteins. $(1-T_{fne})$ and $(1-T_{fno})$ give thermal distances to the melting of bulk ice. These values can simply be read off from the MDs or DMDs. T_{fno} is the start of the temperature independent part in the MD; T_{fne} marks the end temperature of this section. *HeR* gives information on that the surface of the protein molecule to what extent can be considered as heterogeneous (disordered).

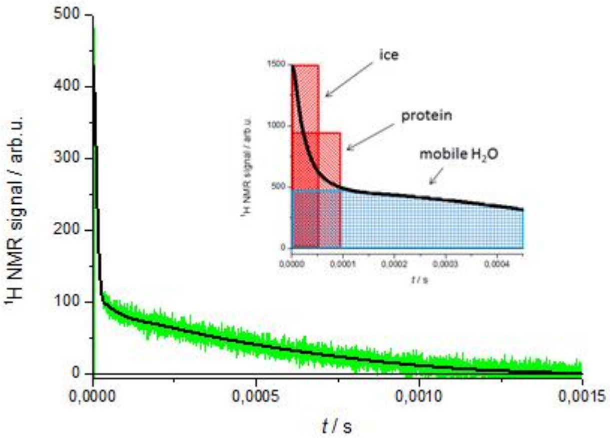

Figure 1S. ^{1}H-NMR signal intensity as function of time measured for TC5b_N1R at T = -30 °C (green data). As T_2 (spin-spin relaxation time) of the component ^{1}Hs situated in ice, liquid water and protein are different, their spectral contribution can be deconvoluted and determined. Unlike protons of the mobile water relaxing slowly, those of the ice (< 50 μs) and the protein (< 100-200 μs) decay fast. Thus, the FID component of the freely moving mobile H_2O could be studied in a large temperature window (-80 °C < T < 0°C) once „extracted" from the ensemble (recorded FID) of those components decaying slowly (> 100 μs).

* $R = 8.314\cdot10^{-23}\ \mathrm{J\ K^{-1}}$ is the molar gas constant, T is the absolute temperature, $k_B = 1.381\cdot10^{-23}\ \mathrm{J\ K^{-1}}$ is the Boltzmann's constant, $N_A = 6.02\cdot10^{23}$ is the Avogardo constant.

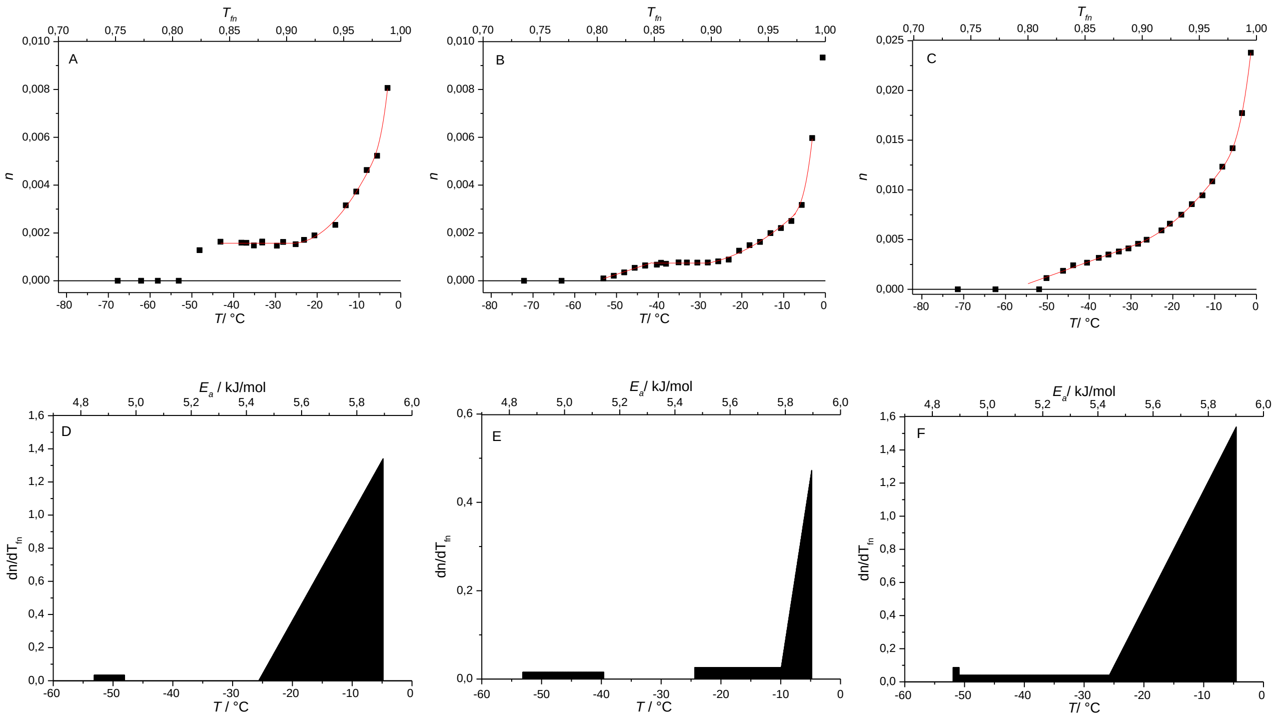

Figure 2S. Melting diagram of TC5b **A**) in neutral condition, **B**) in acidic condition and **C**) for TC5b_N1R dissolved in water at pH = 6.9. Continuous red line was obtained by polynomial fitting of the raw FID data points of the wide-line ^{1}H-NMR spectra ($n = A + B(T_{\mathrm{fn}} - T_{\mathrm{fne}}) + C(T_{\mathrm{fn}} - T_{\mathrm{fn2}})^2 + D(T_{\mathrm{fn}} - T_{\mathrm{fn3}})^3$). Horizontal axis gives temperature (-80 < T(°C) < 0) and normalized fundamental temperature (T_{fn}), while vertical axis measures n (number of mobile H_2Os) from signal intensities. Differential quotient of the fitted polynomial of TC5b **D**) in neutral and **E**) acidic condition as well as **F**) for TC5b_N1R at neutral pH. Horizontal axis stands for the potential energy barrier while the vertical for the differential quotient (dn/dT_{fn}).

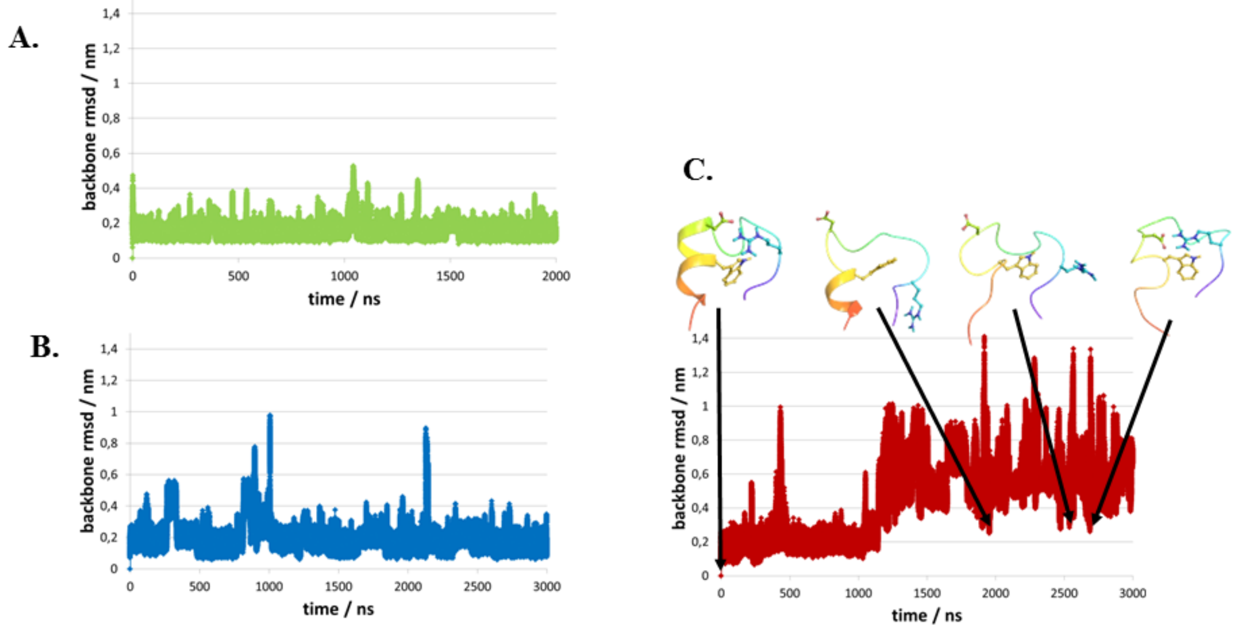

Figure 3S. Backbone fluctuation (expressed in rmsd from the starting molecular structure) of the well folded **A**) TC5b at pH=7 (top/green), **B**) gently unfolded/folded TC5b(H+) (middle/blue) and **C**) of the poorly folded TC5b_N1R (bottom/red). Selected examples above the plot illustrate that folded-like main-chain conformation with the Trp buried in the restored hydrophobic core was re-sampled even in the final 2-3 μs.

A.

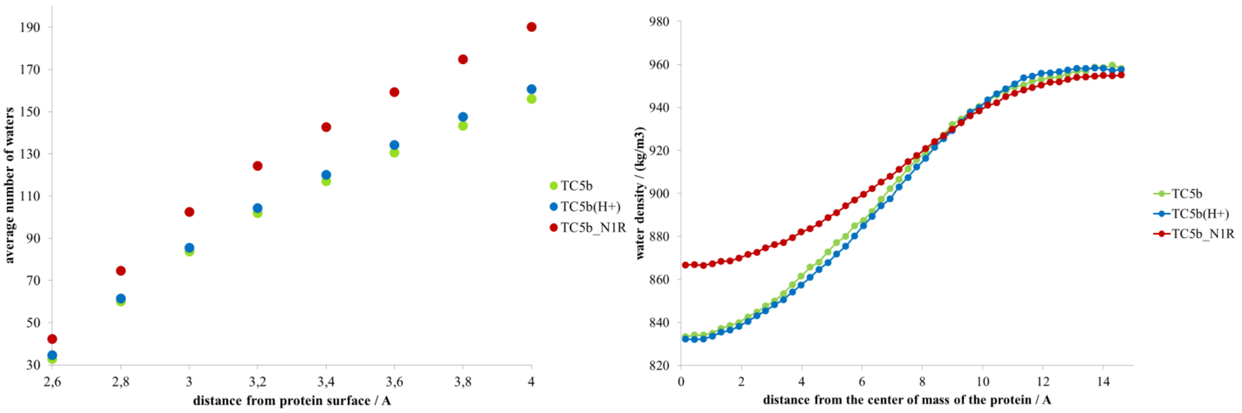

B.

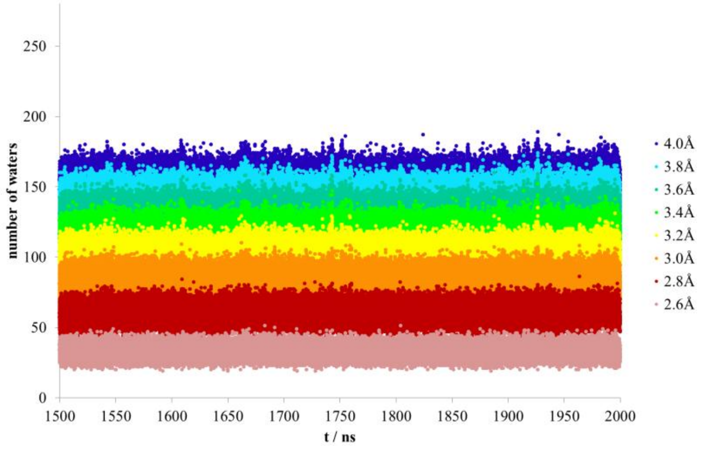

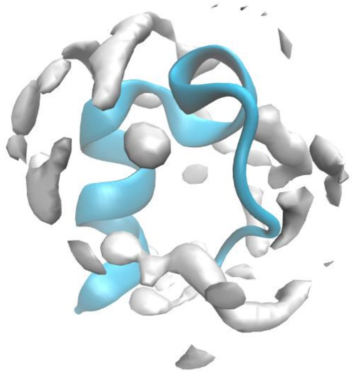

C.

D.

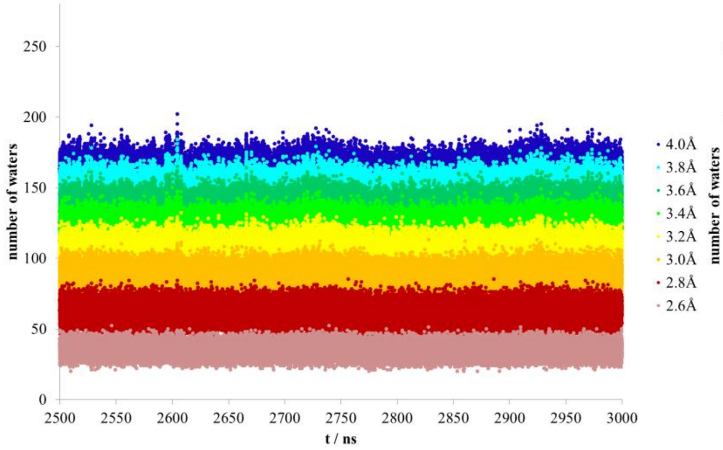

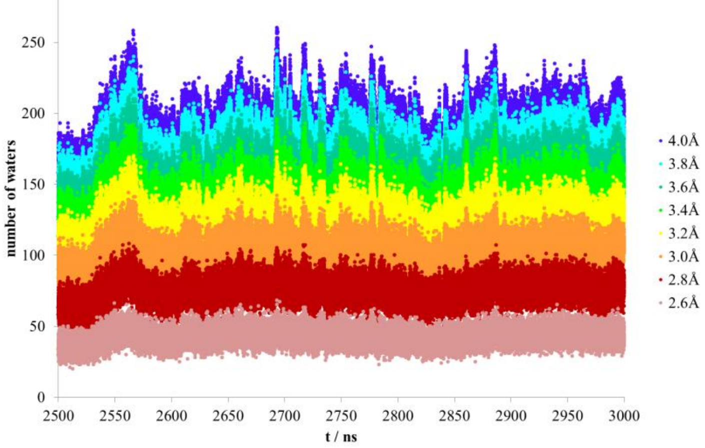

Figure 4S. A) Average number of waters at a given distance of the protein surface (any atom of water closer than the given distance to any atom of the protein) and average water density at a given distance from the center-of-mass of the protein, as a function of the distance (in Å). The number of waters near the protein along the last 500 ns of the MDS trajectory in case of **B**) TC5b at pH=7, **C**) TC5b(H+) and **D**) TC5b_N1R. Next to graph **B)** a VOLMAP representation of the water density around the mid-structure of cluster1 of TC5b is shown (created using VMD [10])

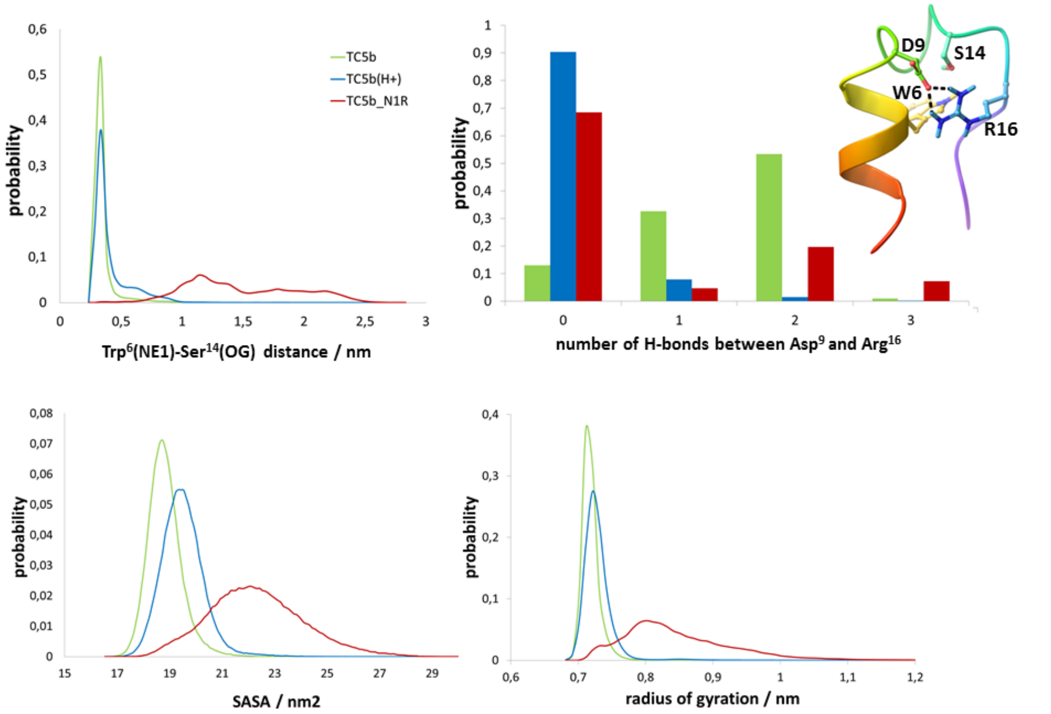

Figure 5S. Structural features of the equilibrium ensembles of TC5b (green), TC5b(H+) (blue) and TC5b_N1R (red) derived from MDS. **Top:** The probability distribution of the Trp^{6}↔Ser^{14} distance and that of the Asp^{9}↔Arg^{16} salt bridge. **Bottom**: The probability distribution of the solvent accessible surface, SASA, and the radius of gyration.

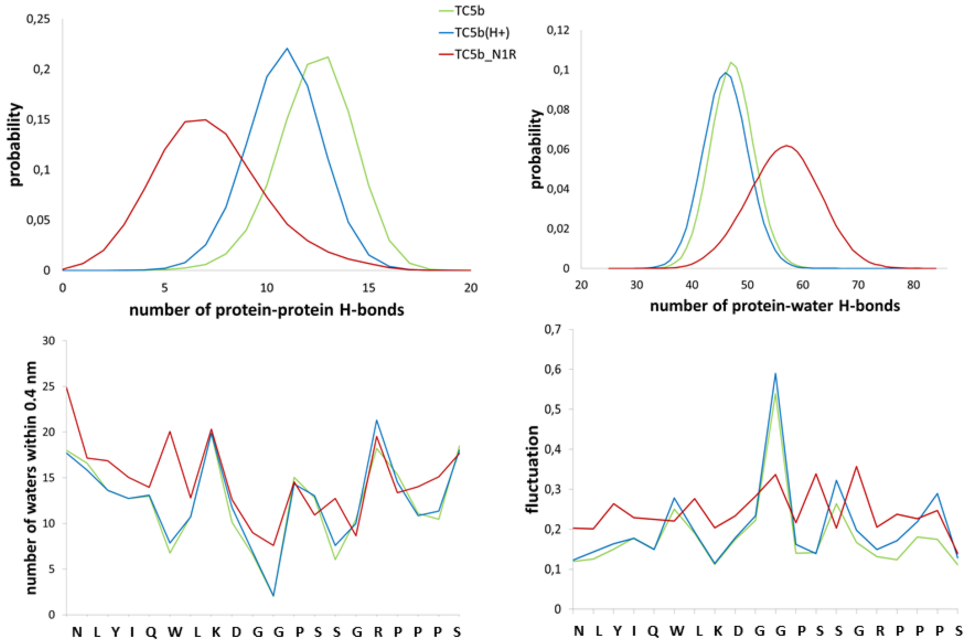

Figure 6S. Top: The probability distribution of the number of intramolecular and protein-water H-bonds formed in the equilibrium ensembles of TC5b (green), TC5b(H+) (blue) and TC5b_N1R (red) derived from MDS. **Bottom:** The average number of waters within 4 Å of each residue, $\langle N_w \rangle$, and its fluctuation: $\sqrt{\langle N_w^2 \rangle - \langle N_w \rangle^2} / \langle N_w \rangle$.

References:

(1) Tompa, K. *et al*. Hydrogen Mobility and Protein–Water Interactions in Proteins in the Solid State. *ChemPhysChem* **18**, 677-682 (2017).

(2) Tompa, K. et al. Interfacial water at protein surfaces: wide-line NMR and DSC characterization of hydration in ubiquitin solutions. Biophys. J. **96**, 2789-2798 (2009).

(3) Tompa, K., Bokor, M., Verebélyi, T., Tompa, P. Water rotation barriers on protein molecular surfaces. *Chemical Physics* **448**, 15-25 (2015).

(4) Tompa, K., Bokor, M., Tompa, P. PART II. Chapter 12. Hydration of intrinsically disordered proteins from wide-line NMR in *Instrumental Analysis of Intrinsically Disordered Proteins: Assessing Structure and Conformation* (ed. Uversky, V. N., Longhi, S.) 345–368 (New Jersey, 2010).

(5) Tompa, K., Bokor, M., Tompa, P. Wide-line NMR and Protein Hydration in *Intrinsically Disordered Protein Analysis* (ed. Uversky, N. V., Dunker, A. K.) 167–196 (New Jersey, 2012).

(6) Tantos, A *et al*. Multiple fuzzy interactions in the moonlighting function of thymosin-β4. *Intrinsically Disordered Proteins* **1**, e26204, 10.4161/idp.26204 (2013)

(7) Kittel, C., Kroemer, H. Appendix B. Temperature Scales in *Thermal Physics* (ed. Freeman, W. H.) 445 (San Francisco, 1980).

(8) McGee, T. D. *Principles and methods of temperature measurement* 32 (New Jersey, 1988)

(9) Haynes, W. M. (Ed.-in-Chief), Section 6: Fluid properties (6-151) in *CRC Handbook of Chemistry and Physics*, 95th edition (Boca Raton, 2014-2015).

(10) Humphrey, W., Dalke, A., Schulten, K. VMD - Visual Molecular Dynamics, *J. Molec. Graphics* **14**, 33-38 (1996) http://www.ks.uiuc.edu/Research/vmd/
